# Profile of partners who completed HIV testing and received a new HIV diagnosis in Ukraine’s HIV index testing program: a retrospective cohort study to inform program improvement

**DOI:** 10.1186/s12879-023-08281-1

**Published:** 2023-05-05

**Authors:** Nancy Puttkammer, Alyona Ihnatiuk, Anna Shapoval, Anna Kazanzhy, Andrew Secor, Solmaz Shotorbani, Misti McDowell, Matthew Golden

**Affiliations:** 1 Department of Global Health, International Training and Education Center for Health (I-TECH), University of Washington, 325 Ninth Ave, Box # 359932, Seattle, WA 98104 USA; 2 International Training and Education Center for Health (I-TECH), 29 Obolonska St., Office 506, Kiev, 04071 Ukraine; 3 Department of Global Health, University of Washington, 325 Ninth Ave, Box # 359932, Seattle, WA 98104 USA

**Keywords:** Ukraine, HIV Testing, Partner notification, Sexual and gender minorities, People who inject drugs

## Abstract

**Background:**

Approximately one-third of people living with HIV in Ukraine are unaware of their HIV status. Index testing (IT) is an evidence-based HIV testing strategy that supports voluntary notification of partners with HIV risk, so they can receive HIV testing, prevention, and treatment services.

**Methods:**

Ukraine scaled up IT services in 2019. This observational study of Ukraine’s IT program covered 39 health facilities located in 11 regions with high HIV burden. The study used routine program data from January—December 2020 to describe the profile of named partners and explore index client (IC) and partner factors associated with two outcomes: 1) completing testing; and 2) HIV case finding. Analysis used descriptive statistics and multilevel linear mixed regression models.

**Results:**

The study included 8,448 named partners, of whom 6,959 had unknown HIV status. Among them,72.2% completed HIV testing and 19.4% of those tested were newly diagnosed with HIV. Two-thirds of all new cases were among partners of ICs who were recently diagnosed and enrolled in care (< 6 months), while one third were among partners of established ICs. In adjusted analysis, partners of ICs with unsuppressed HIV viral load (VL) were less likely to complete HIV testing (adjusted odds ratio [aOR] = 0.11, *p* < 0.001), but more likely to receive a new HIV diagnosis (aOR = 1.92, *p* < 0.001). Partners of ICs who cited injection drug use or having a known HIV + partner as their own reason for testing were more likely to receive a new HIV diagnosis (aOR = 1.32, *p* = 0.04 and aOR = 1.71, *p* < 0.001 respectively). Involving providers in the partner notification process was associated with completed testing (aOR = 1.76, *p* = 0.001) and HIV case finding (aOR = 1.64, *p* < 0.01), compared with notification by ICs.

**Conclusion:**

HIV case detection was highest among partners of recently diagnosed ICs, but IT participation among established ICs still yielded an important share of all newly-identified HIV cases. Areas for improvement in Ukraine’s IT program include completing testing for partners of ICs with unsuppressed HIV VL, with history of injection drug use or discordant partnerships. Using intensified follow-up for the sub-groups at risk of incomplete testing may be practical. Greater use of provider-assisted notification could also accelerate HIV case finding.

**Supplementary Information:**

The online version contains supplementary material available at 10.1186/s12879-023-08281-1.

## Introduction

The HIV epidemic in Eastern Europe and Central Asia is growing faster than in any other region of the world [1]. As of 2021, Ukraine had approximately 240,000 people living with HIV – for a prevalence rate of 0.9% among the adult population aged 15–49 years—the second largest number of people living with HIV (PLHIV) in the region after Russia [2, 3]. Also in 2021, UNAIDS estimated that 180,000 of all PLHIV in Ukraine knew their status (75%), considerably lower than the level estimated for Western and Central Europe and North America at the comparable timepoint (91%) [3, 4] and short of UNAIDS target that 95% of all PLHIV know their status by 2030. The Ukraine epidemic is diverse and vulnerable populations face a heavy burden of HIV and lower knowledge of status rates. For example, the HIV prevalence and knowledge of status rates were estimated at 20.9% and 51% among persons who inject drugs (PWID), 3.1% and 58.2% among sex workers, and 3.9% and 72% among for men who have sex with men (MSM) respectively [3].

In December 2016, the World Health Organization (WHO) recommended scale-up of index testing (IT) services, also known as assisted partner notification services, to increase HIV testing in groups at highest risk for HIV [5, 6]. IT programs offer contact tracing services to clients with HIV or other sexually transmitted infections to confidentially notify contacts of their exposure and link them to testing, prevention, and treatment services. In Ukraine, the scale up of a formal IT program began in 39 public health facilities in 2019 through support from the US President’s Emergency Plan for AIDS Relief (PEPFAR). The results of Ukraine’s IT program contribute in several important ways to the global evidence base on HIV testing interventions to achieve HIV epidemic control. First, while numerous studies and reports have examined IT programs in sub-Saharan Africa, the U.S., the U.K, and China, very little data exist on IT programs in Eastern Europe [7–19]. Second, Ukraine’s HIV epidemic includes many PWID, a population for which contemporary IT outcome data are sparse. Third, Ukraine uses a distinctive clinician-led IT model with IT services integrated at every clinical visit for PLHIV engaged in care.

IT program effectiveness depends upon a cascade of offering IT services, accepting participation, naming partners, partners completing testing and linking to appropriate prevention and care services, as well as the underlying HIV prevalence and undiagnosed fraction of PLHIV in the population. Our prior analyses of Ukraine’s IT program found modest participation in the program, high HIV case finding, and excellent linkage of partners to HIV prevention and care. Specifically, the program reported that 51.9% of index clients (ICs) offered IT services chose to enroll, that 87.5% of participating ICs named a single partner only, that 72.2% of partners with unknown status completed HIV testing, that 19.3% of newly-tested partners were diagnosed with HIV, and that 96.6% of all newly diagnosed partners were linked to HIV care and treatment [20]. Notably, the HIV case finding index, or number of newly diagnosed partners per IC, was 0.14 in Ukraine. The program’s productivity was particularly strong among recently diagnosed ICs (case finding index = 0.29) compared with ICs who had been enrolled in care for more than 6 months (case finding index = 0.07). Ukraine’s overall HIV case finding index was comparable of superior to rates observed in the US in 2019 (0.054) [17], the UK in 2018 (0.066) [16], Botswana in 2018–2020 (0.14) [12], and Namibia in 2019–2021 (0.14) [21].

The purpose of the present study was to identify program gaps and guide program performance improvement efforts. We were interested in the efficiency of the program as a public health intervention, and therefore sought evidence about client sub-groups where it could be productive to focus intensive attention in the context of finite health system staffing and resources for the IT program. As such, we were particularly interested in factors associated with lesser versus greater HIV case finding among partners of established ICs, for whom overall HIV case finding was less. The objectives of the present study were to: 1) describe the proportion of named partners completing HIV testing and explore IC and partner factors associated with this outcome; and 2) describe the proportion of tested partners receiving a new HIV diagnosis and explore IC and partner factors associated with this outcome. We sought to detect the profile of clients who were less likely to complete HIV testing, as well as who were more likely to be newly diagnosed with HIV, so that this information could be used to guide program quality improvement efforts.

## Methods

### Setting and program overview

The International Training and Education Center for Health (I-TECH) at the University of Washington (UW) assisted the Ukraine Ministry of Health (MOH) in IT program implementation from September 2019 to September 2021, with funding from the US Health Resources and Services Administration (HRSA). The US Centers for Disease Control and Prevention (CDC) worked with I-TECH and the MOH to establish the technical direction of Ukraine’s IT program in alignment with PEPFAR programs globally. While Ukraine had in place a national policy endorsing IT services as part of the national package of primary care services prior to our intervention, IT services were not routinely implemented prior to September 2019. At that time, I-TECH developed standard operating procedures, trained health workers on the program, and developed and implemented monitoring and evaluation tools to track IT program implementation and outcomes. During September to December 2019, I-TECH mentors provided training and supported the routinization of IT program implementation at the 39 health facilities; the standard operating procedures for the IT program remained consistent through the timeframe reported in the present study.

Ukraine’s IT program relies on clinicians, including infectious disease doctors, nurses, and psychologists, rather than dedicated non-clinical staff [20]. IT services are integrated within the routine HIV clinical workflow, as prompted by an electronic health record (EHR) system during each clinical visit. As such, the program focuses on partner tracing among both recently diagnosed and established HIV clients. The IT program is voluntary, and ICs can select their preferred mode of partner notification, including: 1) client notification (IC notifies the partner); 2) provider notification (where health workers notify partners directly without mentioning the identity of the IC); 3) joint notification (where the IC and health worker together notify the partner); or 4) contract notification (where health workers notify partners if the IC is unable to do so within a planned timeframe). For each named partner, health workers assess for risk of intimate partner violence (IPV). Ukraine’s HIV testing and diagnostic protocol uses serial rapid tests and allows for same-day HIV diagnosis for most clients [22].

### Study design and facility sample

This observational study took place in 39 health facilities located in 11 out of the 12 PEPFAR-prioritized oblasts (regions), high-burden regions where approximately 75% of PLHIV and 54% of the population of Ukraine reside [23]. The 39 health facilities served the largest numbers of HIV clients for care and treatment services within the 11 oblasts. As of December 2020, 49,693 PLHIV (including children) received HIV-related services at these health facilities. The cohort of clients included in the study were partners named by ICs who participated in IT services during January to December 2020.

### Data sources and data collection methods

The study used routinely collected program data from two data sources: 1) the Socially Important Diseases Medical Information System (SID MIS), an electronic health record system used by all health facilities providing IT services as part of routine HIV clinical services; and 2) an IT services program register. Both tools collected information on IC age and sex, partner type(s), IT partner notification mode, and partner age group, sex, and HIV testing status. All partners had at least 60 days of follow-up time to observe their HIV testing outcomes.

### Study population

Ukraine’s HIV IT program is considered a routine, primary care service in all health facilities offering IT services. As such, study participants included all ICs at the 39 health facilities who accepted IT services during the time frame of interest. The study included all named partners of the ICs. Partners were categorized based on HIV exposure risk as either sexual partners, needle-sharing partners, or biological children of mothers living with HIV, in accordance with PEPFAR Monitoring, Evaluation and Reporting (MER) guidance [24].

### Study measures

There were two outcomes of interest: 1) completing HIV testing within 60 days, among named partners with unknown HIV status; and 2) receiving a new diagnosis of HIV infection, among partners who completed HIV testing. Our analysis of the testing outcome excluded partners already known to be living with HIV (whether already linked to care or not) and those for whom the IC reported an IPV concern. We considered partners’ HIV testing disposition within 60 days of being named by an IC, since testing within this time frame could be attributed to the IT program rather than the partner’s independent initiative, and since 93.6% of IT partners who completed testing did so within 60 days. IC characteristics of interest included sex, age, reason for testing (including MSM or PWID status, having a known HIV + partner, and being pregnant for women), number of named partners (grouped as one or multiple), time since HIV diagnosis (grouped as < 6 months, called “recently-diagnosed” vs. ≥ 6 months, called “established” ICs), and HIV viral suppression status (grouped as on ART with suppressed VL, unsuppressed [including those not yet on ART or on ART with a documented unsuppressed VL], or on ART but with no VL data available from 365 days before to 2 months after IT acceptance date). HIV viral suppression was defined as having a VL < 1,000 copies/ml. Partner characteristics included sex, age, HIV transmission risk category (grouped as sexual partner, needle-sharing partner, or biological child), and partner notification mode (grouped as client, provider, joint, or contract notification).

### Data analysis

We first de-duplicated partner records using unique identifiers from the SID MIS data system, a process taking place in several steps, linking records for the same partner named by different ICs through a unique identifier in the SID MIS system. We used the earliest HIV positive test result or the most recent HIV negative test result as the definitive HIV status outcome for each de-duplicated partner. In our analytic sample, there were several partners named by two different ICs (*n* = 23); for these, we randomly selected one of the two IC cases as the reference case for our exploration of IC characteristics associated with the IT outcomes.

We used descriptive statistics to summarize partner characteristics and their HIV testing disposition, assessing frequencies for categorical variables and measures of central tendency and dispersion for continuous variables. We first considered bivariable models and included those factors with significant associations (*p* < 0.05 using Wald test) in our final multivariable models. For each analysis, we used mixed effects logistic regression models, which treated health facility and ICs as random effects (to address nesting of observations on partners within health facilities and ICs) [25]. Results with *p*-values < 0.05 were considered statistically significant in our exploratory analysis. We performed analyses using R Statistical Software (Foundation for Statistical Computing, Vienna, Austria) and Stata 15.0 (College Station, TX, USA). To identify factors specifically relevant to testing completion and HIV case finding for partners of established ICs, we carried out the same analysis with stratification by partners of recently diagnosed and established ICs.

### Study ethics

The Ethics Committee of the Ukraine MOH Center for Public Health, the UW Human Subjects Division, and the CDC reviewed the study protocol. All three approved the study as a program evaluation with minimal risk to human subjects and waived the requirement for client consent to participate based on the secondary use of routinely collected, deidentified client data.

## Results

**Fig. 1:**
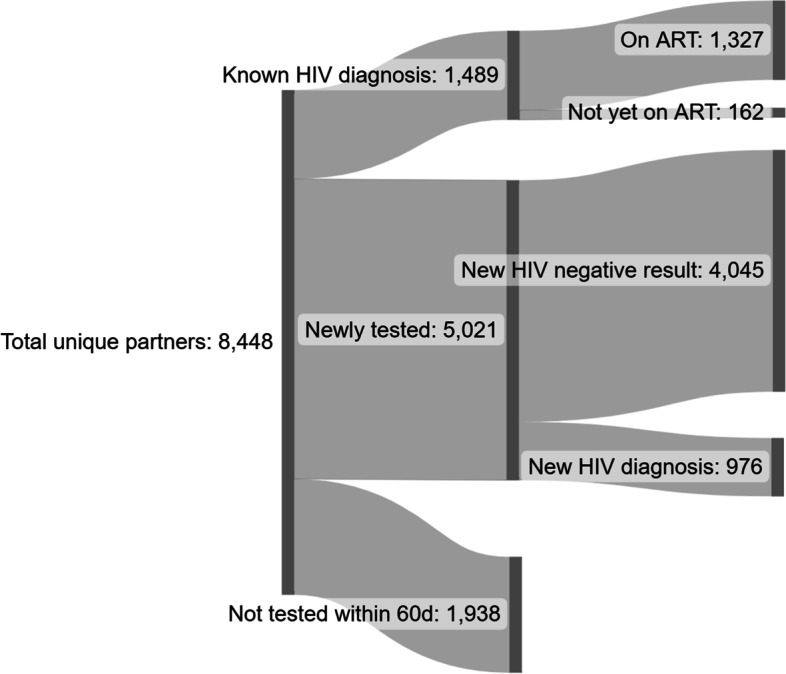
IT program participants at 39 health facilities in Ukraine, January – December 2020

At the selected 39 facilities, 7,408 ICs named at least one partner, for a total of 8,448 unique partners (Fig. 1). About one third were partners of ICs with recent diagnoses (i.e., within six months, 34.7%) while two-thirds were partners of ICs who had established HIV diagnoses (65.3%). Overall, 82.4% of named partners had an unknown HIV status, with a higher share among partners of recently diagnosed ICs compared with partners of established ICs (87.7% vs. 79.5%). Twenty-six partners (0.3%) were cases with IPV concern who were not followed for notification and testing. A total of 5,021 partners completed HIV testing within 60 days of being named (72.2% of all partners with unknown HIV status), with similar rates of testing completion among partners of recently diagnosed and established ICs (73.0% vs. 71.7%). Among the newly tested, 976 (19.4%) were newly diagnosed with HIV, with a three-fold higher level among partners of recently diagnosed ICs compared to established ICs (34.7% vs. 10.3%) (Fig. 1). Among the newly identified HIV cases, 6 had previously tested negative through the IT program. There were no cases of IPV because of IT program participation as reported by ICs (results not shown).

### Partner characteristics and HIV testing disposition

Table 1 shows the characteristics of the 8,448 named partners by their HIV testing disposition. Overall, 52.0% of partners were male, and mean partner age was 39.1 years (SD = 8.9). Most were sexual partners (88.7%), 4.6% were needle-sharing partners, and 6.7% were biological children. Client notification was most common (60.6%), followed by joint notification (16.0%), provider notification (6.6%), and contract notification (0.8%). Partners were evenly divided by the HIV viral suppression status of the ICs who named them: 34.8% were partners of ICs with suppressed HIV VL, 30.7% were partners of ICs with unsuppressed VL (including ICs who had not yet started ART), and 34.4% were partners of ICs on ART but without VL results available.

**Table 1:** Index client, partner, and partnership characteristics, by partner testing outcome (*n* = 8,448)

**Characteristics n (row %)**	**All named partners n (% of total)**	**Partners already known HIV + and enrolled on ART n (row %)**	**Partners already known HIV + but not already linked to ART n (row %)**	**Partners newly diagnosed with HIV n (row %)**	**Partners newly testing HIV negative n (row %)**	**Partners not tested within 60 days n (row %)**
***Total***	**8,448**	**1,322 (15.7)**	**167 (1.9)**	**976 (11.6)**	**4,045 (47.9)**	**1,938 (22.9)**
***Index client characteristics ***						
***IC Sex***						
Male	4,169 (49.3)	708 (17.0)	82 (2.0)	507 (12.2)	1,995 (47.9)	877 (21.0)
Female	4,279 (50.7)	619 (14.5)	80 (1.9)	469 (11.0)	2,050 (47.9)	1,061 (24.8)
***Age**** (mean, sd)*	39.1 (SD = 8.9)	40.6 (8.6)	38.9 (8.3)	39.4 (9.1)	38.9 (9.0)	38.5 (8.9)
***IC reason for testing at own HIV diagnosis***						
PWID	1,330 (15.7)	85 (6.4)	31 (2.3)	242 (18.2)	820 (61.7)	152 (11.4)
MSM	153 (1.8)	26 (17.0)	1 (0.7)	22 (14.4)	69 (45.1)	35 (22.9)
HIV + partner	1,056 (12.5)	174 (16.5)	49 (4.6)	172 (16.3)	503 (47.6)	158 (15.0)
Pregnancy	941 (11.1)	36 (3.8)	22 (2.3)	111 (11.8)	613 (65.1)	159 (16.9)
***IC recency of diagnosis***^***a***^						
Recent diagnosis (< = 6 mos)	2,931 (34.7)	280 (9.6)	80 (2.7)	651 (22.2)	1225 (41.8)	695 (23.7)
Established diagnosis (> 6 mos)	5,517 (65.3)	1042 (18.9)	87 (1.6)	325 (5.9)	2820 (51.1)	1243 (22.5)
***IC viral suppression status***						
On ART with VL < = 1000	2,943 (34.8)	173 (5.9)	49 (1.7)	261 (8.9)	2221 (75.5)	239 (8.1)
Not on ART or on ART with VL > 1000	2,596 (30.7)	327 (12.6)	74 (2.9)	479 (18.5)	1016 (39.1)	700 (27.0)
On ART with no VL available	2,909 (34.4)	822 (28.3)	44 (1.5)	236 (8.1)	808 (27.8)	999 (34.3)
***Partner characteristics***						
***Partner sex***						
Male	4,390 (52.0)	664 (15.1)	92 (2.1)	510 (11.6)	2077 (47.3)	1047 (23.8)
Female	4,058 (48.0)	663 (16.3)	70 (1.7)	466 (11.5)	1968 (48.5)	891 (22.0)
***Age**** (mean, sd)*^*b*^	37.2 (SD = 12.2)	41.2 (8.2)	37.4 (8.7)	39.1 (10.4)	36.2 (13.0)	36.7 (12.7)
***Partner notification method***						
Client notification	5,119 (60.6)	-	141 (2.8)	627 (12.2)	2,894 (56.5)	1457 (28.5)
Provider notification	554 (6.6)	-	7 (1.3)	122 (22.0)	293 (52.9)	132 (23.8)
Contract notification	70 (0.8)	-	4 (5.7)	8 (11.4)	41 (58.6)	17 (24.3)
Joint notification	1351 (16.0)	-	10 (0.7)	219 (16.2)	817 (60.5)	305 (22.6)
***Type of partnership***						
Sexual	7,490 (88.7)	1268 (16.9)	141 (1.9)	890 (11.9)	3,448 (46.0)	1743 (23.3)
Needle sharing	391 (4.6)	53 (13.6)	18 (4.6)	70 (17.9)	214 (54.7)	36 (9.2)
Biological child	567 (6.7)	6 (1.1)	3 (0.5)	16 (2.8)	383 (67.5)	159 (28.0)

*PWID* persons who injects drugs (indicating current or past injection drug use), *MSM* Man having sex with men, *VL* HIV viral load, *ART* Antiretroviral therapy

^a^Recently diagnosed clients are ICs with up to six months from HIV diagnosis to date when IT was accepted, while established clients are ICs with more than 6 months from HIV diagnosis to date when IT was accepted

^b^Partner age data only available among 6,445 partners (76.3% of named partners)

### Factors associated with completion of partner testing

Table 2 shows factors associated with completion of HIV testing among partners with unknown status (*n* = 6,932). Completion of testing was highest among those with joint notification (77.3%) and provider notification (75.9%) and lowest among those with client notification (70.7%). Factors not associated with testing completion in bivariable analyses included IC age, IC MSM status, IC recency of HIV diagnosis, and the number of partners reported by the IC. In adjusted analysis, factors associated with greater likelihood of partner testing included provider notification (adjusted odds ratio [aOR] = 1.76, *p* = 0.001) or joint notification (aOR = 1.56, *p* < 0.001) as compared to client notification. Partners of ICs with unsuppressed VL or with no recent VL were less likely to complete testing (aOR = 0.11 and aOR = 0.05 respectively, both *p* < 0.001). Partners of male ICs were more likely to complete testing (aOR = 1.47, *p* = 0.02) as were partners with ICs citing PWID (aOR = 5.56, *p* < 0.001), having a known HIV + partner (aOR = 3.47, *p* < 0.001), or pregnancy (aOR = 3.14, *p* < 0.001) as their own reason for testing. In stratified analysis, factors associated with testing among partners of established ICs were similar to the overall analysis; however, partners of male ICs were no more likely to complete testing than partners of female ICs in this sub-group (Supplemental Table 1).

**Table 2:** Factors associated with partner completing testing (*n* = 6,932 partners with unknown status^a^)

Variable (reference category)	Comparison group	Bivariable model			Multivariable model		
		**OR**	**95% CI**	***p*-value**	**aOR**	**95% CI**	***p*-value**
IC sex (ref = female)	Male	1.28	(1.09, 1.50)	0.002	1.47	(1.06, 2.05)	0.022
IC age (continuous)	Each 5-year increase	1.03	(0.99, 1.08)	0.19			
IC reason for testing at own HIV diagnosis (ref = no)	PWID	4.28	(3.24, 5.65)	< 0.001	5.56	(3.98, 7.75)	< 0.001
	MSM	1.35	(0.76, 2.39)	0.31			
	HIV + partner	2.25	(1.71, 2.95)	< 0.001	3.47	(2.56, 4.68)	< 0.001
	Pregnancy	2.42	(1.84, 3.19)	< 0.001	3.14	(2.30, 4.28)	< 0.001
IC number of partners (ref = one)	Multiple	0.84	(0.69, 1.04)	0.10			
IC recent HIV diagnosis (ref = within 6 months)	Established IC (> 6 months)	0.96	(0.81, 1.14)	0.66			
IC HIV VL status (ref = VL < = 1000)	Unsuppressed	0.11	(0.08, 0.16)	< 0.001	0.11	(0.08, 0.15)	< 0.001
	VL not available	0.04	(0.03, 0.06)	< 0.001	0.05	(0.04, 0.08)	< 0.001
Partner sex (ref = female)	Male (ref = female)	0.85	(0.73, 1.00)	0.04	1.03	(0.75, 1.40)	0.87
Partner type (ref = sexual)	Needle-sharing	2.86	(1.79, 4.56)	< 0.001	0.86	(0.49, 1.53)	0.61
	Child	1.14	(0.86, 1.51)	0.36	1.25	(0.91, 1.73)	0.17
Mode of partner notification (ref = client notification)	Contract	0.99	(0.44, 2.23)	0.97	0.92	(0.40, 2.13)	0.85
	Provider	1.55	(1.14, 2.10)	0.005	1.76	(1.28, 2.43)	0.001
	Joint	1.55	(1.24, 1.93)	< 0.001	1.56	(1.23, 1.97)	< 0.001

*OR* Odds ratio, *aOR* Adjusted odds ratio, *CI* Confidence interval, *IC* Index client, *ART* Antiretroviral therapy, *PWID* Person with current or past injection drug use, *MSM* Man having sex with men, *VL* HIV viral load

^a^Excludes 27 partners with IPV concern reported. Data on partner age was missing for 67.7% of partners who did not complete testing, so this factor was excluded from the analysis. Results based on mixed effects logistic regression models, which treated health facility and ICs as random effects (to address nesting of observations on partners within health facilities and ICs). Health facilities in the sample had between 12 and 645 partners (mean = 161) and ICs had between one and 14 partners (mean = 1.1)

### Factors associated with HIV case finding

Table 3 shows factors associated with HIV case finding among 5,021 partners newly tested through the IT program. Factors not associated with HIV case finding in bivariable analyses included IC sex, IC MSM status, and the number of partners reported by the IC (Table 3). In adjusted analysis, partners of ICs with unsuppressed HIV VL carried a nearly two-fold risk of HIV diagnosis compared with partners of ICs with viral suppression (aOR = 1.92, *p* < 0.001). Provider notification was also strongly associated with HIV case finding (aOR = 1.64, *p* < 0.01). Older partners were slightly more likely to receive a new HIV diagnosis (aOR = 1.06 for each additional 5 years of age, *p* = 0.06), as were partners of ICs who cited PWID or having known HIV + partner as their reason for testing (aOR = 1.32, *p* = 0.04 and aOR = 1.71, *p* < 0.001 respectively) (Table 3). In contrast, partners of established ICs and partners who were biological children were less likely to receive a new HIV diagnosis (aOR = 0.22 and aOR = 0.11 respectively, both *p* < 0.001). In stratified analysis, factors associated with HIV case finding among partners of established ICs included using provider or contract notification and IC’s unsuppressed VL, but not ICs having an unavailable VL. Being a partner of an IC with a HIV + partner as reason for testing was associated with HIV case finding in this sub-group but being a partner of an IC with PWID as reason for testing was not (Supplemental Table 2).

**Table 3:** Factors associated with partner’s HIV positive diagnosis (*n* = 5,021 newly tested partners)

Variable (reference category)	Comparison group	Bivariable model			Multivariable model		
		**OR**	**95% CI**	***p*-value**	**aOR**	**95% CI**	***p*-value**
IC sex (ref = female)	Male	1.12	(0.95, 1.31)	0.18			
IC age (continuous)	Each 5-year increase	1.05	(1.00, 1.10)	0.04	1.00	(0.93, 1.07)	0.92
IC reason for testing at own HIV diagnosis (ref = no)	PWID	1.34	(1.10, 1.63)	0.004	1.32	(1.01, 1.71)	0.04
	MSM	1.40	(0.79, 2.49)	0.25			
	HIV + partner	1.56	(1.24, 1.96)	< 0.001	1.71	(1.29, 2.26)	< 0.001
	Pregnancy	0.69	(0.54, 0.88)	0.003	1.32	(0.98, 1.79)	0.07
IC number of partners (ref = one)	Multiple	1.05	(0.85, 1.30)	0.65			
IC recency of HIV diagnosis (ref = recently diagnosed)	Established IC (> 6 months)	0.16	(0.12, 0.22)	< 0.001	0.22	(0.16, 0.26)	< 0.001
IC HIV viral load (VL) status (ref = VL < = 1000)	Unsuppressed	4.86	(3.62, 6.52)	< 0.001	1.92	(1.44, 2.26)	< 0.001
	VL not available	2.79	(2.14, 3.64)	< 0.001	1.32	(0.99, 1.74)	0.06
Partner sex (ref = female)	Male (ref = female)	1.07	(0.91, 1.26)	0.40			
Partner age (continuous	Each 5-year increase	1.14	(1.09, 1.18)	< 0.001	1.06	(1.00, 1.13)	0.06
Partner type (ref = sexual)	Needle-sharing	1.46	(1.01, 2.10)	0.04	1.19	(0.78, 1.83)	0.42
	Child	0.11	(0.06, 0.20)	< 0.001	0.11	(0.05, 0.26)	< 0.001
Mode of partner notification (ref = client notification)	Contract	0.89	(0.37, 2.10)	0.78	0.81	(0.30, 2.18)	0.68
	Provider	1.70	(1.28, 2.27)	< 0.001	1.64	(1.18, 2.28)	< 0.01
	Joint	1.09	(0.87, 1.35)	0.45	0.98	(0.77, 1.26)	0.90

*OR* Odds ratio, *aOR* Adjusted odds ratio, *CI* Confidence interval, *IC* Index client, *ART* Antiretroviral therapy, *PWID* Person with current or past injection drug use, *MSM* Man having sex with men, *VL* HIV viral load; *Excludes 27 partners with IPV concern reported. Results based on mixed effects logistic regression models, which treated health facility and ICs as random effects (to address nesting of observations on partners within health facilities and ICs). Health facilities in the sample had between 8 and 476 partners (mean = 117) and ICs had between one and 14 partners (mean = 1.1)

Table 4 presents a visual summary of the relationship between partner characteristics and both IT cascade outcomes together. Of note, in adjusted analyses, partners of established ICs and partners who were biological children were equally likely to complete HIV testing, but much less likely to be newly diagnosed with HIV. Partners of ICs with unsuppressed VL results were much less likely to complete testing and more likely to be newly diagnosed with HIV. Partners of ICs with unknown VL results were also less likely to complete testing but did not have an excess risk of new HIV diagnosis. Provider notification was associated with both greater likelihood of completing testing and greater likelihood of new HIV diagnosis.

**Table 4:**
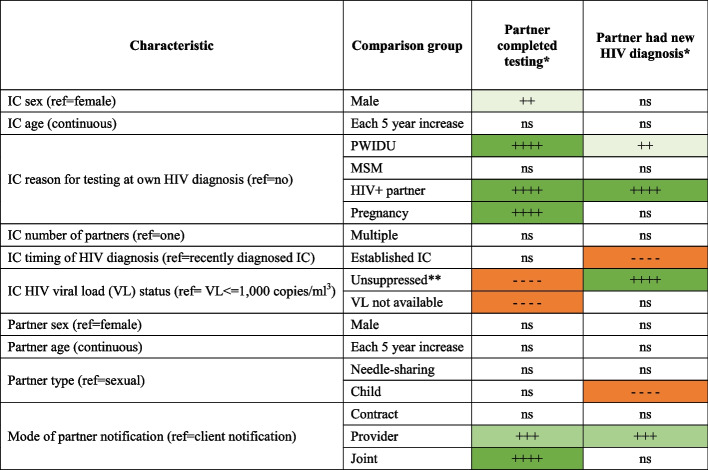
Directions of association for IT program cascade outcomes

*Ns* not statistically significant,*IC* index client

^*^Results based on mixed effects logistic regression models, which treated health facility and ICs as random effects (to address nesting of observations on partners within health facilities and ICs)

^**^Unsuppressed category includes ICs not on ART as well as those with documented VL > 1,000 copies/ml^3^)

Key

## Discussion

Our evaluation provides a detailed profile of ICs and named partners in the IT program at 39 health facilities in 11 high HIV burden regions of Ukraine. In our multivariable models, completed HIV testing among named partners was strongly positively associated with several IC characteristics, including higher age, being male, injection drug use, MSM status, pregnancy, having an HIV-positive partner, and suppressed viral load status. HIV case finding among named partners was also strongly positively associated with several IC characteristics, including recency of HIV diagnosis (i.e., IC having tested HIV-positive within the past six months), injection drug use, having an HIV-positive partner, and unsuppressed viral load, while IC pregnancy was only weakly associated with HIV case finding, and testing of biological children was less likely to yield HIV case finding compared to other partner types. In addition, provider-assisted partner notification methods were associated with greater likelihood of completed HIV testing and case finding among named partners.

Implementation science research in IT programs has demonstrated the value of understanding factors associated with increased HIV testing and case finding [13]. Our research revealed potential areas for optimizing IT services in Ukraine, including promotion of provider-assisted notification to increase HIV testing and case detection, a finding substantiated by the literature. A meta-analysis comprising over 5,000 patients in both high resource and resource limited settings found that provider-assisted partner services resulted in a 1.5-fold increase in HIV testing over passive referral by clients [26], and a recent randomized trial in Malawi that compared social network testing using contract notification to passive client referral found a 1.9-fold increase in HIV case finding [27]. Despite the demonstrated benefits of provider-assisted notification, however, our data showed that ICs chose provider-assisted notification less than 25% of the time, suggesting that additional intervention or education for both providers and ICs on the benefits of provider-assisted notification may improve HIV testing and case detection.

Our results also suggest that service provision could be optimized through prioritized intervention among groups we identified as having higher HIV testing and case detection rates, a common practice among many HIV testing programs globally, including an exclusive focus among many IT programs on recently diagnosed ICs given the typically higher HIV case finding. However, there is debate in HIV testing programs about the benefits of targeting testing to only those sub-groups most likely to produce new HIV case finding. In a commentary on targeted testing strategies, Sanders et al. cautioned against strategies that are “penny-wise and pound-foolish if they miss individuals who are likely to transmit to one or more partners” [28]. The potential drawback of targeted services is evidenced in our data, which showed that nearly one-third of all new cases detected among participants were from named partners of established ICs, suggesting that an exclusive focus on newly diagnosed ICs would result in a substantial number of cases being missed.

These potential missed cases due to targeted services, however, need to be balanced against resource limitations. A study of Botswana’s facility-based HIV testing program considered advantages and disadvantages of targeting HIV testing services to those at higher risk of undiagnosed HIV. It found that a risk score algorithm could reduce testing volume and costs per new HIV case, especially important as at the time only 1% of tests yielded newly diagnosed PLHIV [29]. However, the study found that using risk screening to target HIV testing would be more likely to miss younger PLHIV and those with asymptomatic disease, demonstrating an undesirable trade-off since bringing these groups into HIV care and treatment is critical to HIV epidemic control. In Ukraine, while health policy has taken a universal approach to IT services for all PLHIV enrolled in HIV care, with services routinely offered at every clinical visit for both recently diagnosed and established clients, real-world challenges of COVID-19 and war could drive the need for pragmatic trade-offs. Starting in March 2020, Ukraine had several rolling lock-downs due to COVID-19, health facilities were taxed with caring for COVID-19 patients, health workers were diverted to provide COVID-19 related services, and clients were hesitant to present for non-urgent services. Then, starting in February 2022, the Russian invasion of Ukraine has had a devastating impact on health services [30]. More than 30% of Ukrainians have been displaced from their homes at some point during the war, and more than 226 health facilities have been damaged by bombing [31, 32]. Our study suggests that if targeted services are needed in Ukraine due to these issues or other resource limitations, services could be best optimized by focusing on partners of recently diagnosed ICs, partners of established ICs with unsuppressed or unknown VL, and partners of ICs whose cited reasons for testing included injection drug use, having a known HIV-positive partner, or pregnancy. Of note, our finding that MSM status as a reason for testing was not associated with increased case finding may reflect under-reporting and misclassification rather than a true lack of association [33].

### Strengths and limitations

A key strength of the study was the use of robust, routine data for all clients who participated in IT services in the 39 healthcare facilities during the timeframe of the study, giving a comprehensive picture of the real-world outcomes of IT services in Ukraine. Ukraine’s strong, centralized electronic data system allowed for clear and detailed tracking of partner testing and linkage to care within Ukraine’s IT program. The SID MIS system, in use throughout Ukraine since 2016, made it possible to precisely identify partners who had previously tested positive for HIV, and to distinguish them from partners with unknown HIV status, the focus of this study. A limitation of the study was that the routine data sources used in the study lacked key sociodemographic and other information about ICs and partners, such as education, employment, income, internalized stigma, disclosure of HIV status to others, and health services utilization. These unmeasured factors could potentially be useful in classifying partner sub-groups with sub-optimal outcomes along the IT services cascade, who should receive intensified follow-up, so further research would be needed on such factors. We also lacked information about the timing of HIV exposures for each partner. IT program standard operating procedures guided health workers to focus on eliciting partners with potential exposure to HIV in the past 2–3 years. Further information on exposure timing could help in clarifying the optimal time window to use in eliciting partners in the Ukrainian context. As an observational program evaluation, our study’s results must be interpreted as exploratory evidence of associations, not as evidence of causal relationships. Clients classified in various sub-groups may have been systematically different in ways not evident in our data, leading to unobserved confounding. For example, the strong association between provider notification and HIV case finding could reflect that ICs preferentially referred partners for provider notification if they suspected them to have an undiagnosed HIV infection.

## Conclusion

Ukraine’s IT program has an important role in increasing HIV testing and case detection. Our study demonstrated strong HIV case finding through a scaled, routinely implemented IT program in an Eastern European context. Increasing use of provider-assisted notification methods and focusing intensified partner case management for partners of all ICs with unsuppressed viral loads are priorities to advance HIV case finding in Ukraine. Such targeted IT services may be practical to consider in the context of the current war in Ukraine, where the healthcare system is stretched and there are many competing priorities. However, targeted services need to be weighed against the potential for lower testing and case detection among non-targeted groups.

## Supplementary Information

**Additional file 1.**

## Data Availability

The data that support the findings of this study are available from the Public Health Center of the Ministry of Health of Ukraine (PHC), but restrictions apply to the availability of these data, which were used under agreement with PHC for the current study, and so are not publicly available. Data are however available from the authors upon reasonable request and with permission of PHC, by contacting Nancy Puttkammer (nputt@uw.edu).

## Abbreviations

ART: HIV antiretroviral therapy
CDC: US Centers for Disease Control and Prevention
HIV: Human immunodeficiency virus
HRSA: US Health Resources and Services Administration
IC: Index client (client with index HIV infection)
IPV: Intimate partner violence
IT: Index testing
I-TECH: International Training and Education Center for Health
MSM: Men who have sex with men
PHC: Public Health Center of the Ministry of Health of Ukraine
PWID: Persons with history or current injection drug use
VL: HIV viral load
WHO: World Health Organization
