# Supplementary material for: Profile of partners who completed HIV testing and received a new HIV diagnosis in Ukraine’s HIV index testing program: a retrospective cohort study to inform program improvement
Source: BMC Infect Dis. 2023 May 5;23:291. doi: 10.1186/s12879-023-08281-1 (PMC10161989; doi:10.1186/s12879-023-08281-1)
Supplement: Supplementary file 1 — Additional file 1. [file 12879_2023_8281_MOESM1_ESM.docx]

**Supplemental Table 1. Characteristics of ICs and partners associated with partner’s completion of testing, stratified by recency of IC’s HIV diagnosis***

| **Variable (reference category)** | **Comparison group** | **Partners of recently diagnosed ICs (n=2,559)** | | | **Partners of established ICs (n=4,373)** | | |
| --- | --- | --- | --- | --- | --- | --- | --- |
|  |  | **aOR** | **95% CI** | **p-value** | **aOR** | **95% CI** | **p-value** |
| IC sex (ref=female) | Male | 1.63 | (1.06, 2.51) | 0.03 | 0.97 | (0.56, 1.66) | 0.90 |
| IC reason for testing at own HIV diagnosis (ref=no) | PWID | 2.95 | (1.92, 4.52) | <0.001 | 2.95 | (1.92, 4.52) | <0.001 |
|  | HIV+ partner | 1.38 | (0.97, 1.96) | 0.07 | 1.38 | (0.97, 1.96) | 0.07 |
|  | Pregnancy | 1.34 | (0.85, 2.10) | 0.21 | 1.34 | (0.85, 2.10) | 0.21 |
| IC HIV viral load (VL) status (ref= VL<=1000) | Unsuppressed | 0.49 | (0.30, 0.80) | <0.01 | 0.49 | (0.30, 0.80) | 0.01 |
|  | VL not available | 0.73 | (0.43, 1.23) | 0.24 | 0.73 | (0.43, 1.23) | 0.24 |
| Partner sex (ref=female) | Male | 1.15 | (0.77, 1.72) | 0.48 | 1.15 | (0.77, 1.72) | 0.48 |
| Partner type (ref=sexual) | Needle-sharing | 0.97 | (0.46, 2.04) | 0.93 | 0.97 | (0.46, 2.04) | 0.93 |
|  | Child | 1.19 | (0.78, 1.81) | 0.41 | 1.19 | (0.78, 1.81) | 0.41 |
| Mode of partner notification (ref=client notification) | Provider or contract** | 2.10 | (1.34, 3.27) | 0.001 | 2.10 | (1.34, 3.27) | 0.001 |
|  | Joint | 1.92 | (1.37, 2.71) | <0.001 | 1.92 | (1.37, 2.71) | <0.001 |

aOR=Adjusted odds ratio; CI=Confidence interval; IC=Index client; ART=Antiretroviral therapy; PWID=Person with current or past injection drug use; MSM=Man having sex with men; VL=HIV viral load; ^*^Excludes 27 partners with IPV concern reported.

^* *^ Provider and contract notification modes combined due to small cell size for contract mode. Results based on mixed effects logistic regression models, which treated health facility and ICs as random effects (to address nesting of observations on partners within health facilities and ICs).

**Supplemental Table 2. Characteristics of partners associated with a new HIV positive diagnosis, stratified by recency of IC’s HIV diagnosis***

| **Variable (reference category)** | **Comparison group** | **Partners of recently diagnosed ICs (n=1,876)** | | | **Partners of established ICs (n=3,144)** | | |
| --- | --- | --- | --- | --- | --- | --- | --- |
|  |  | **aOR** | **95% CI** | **p-value** | **aOR** | **95% CI** | **p-value** |
| IC age (continuous) | Each 5-year increase | 1.07 | (0.96, 1.19) | 0.22 | 0.92 | (0.83, 1.02) | 0.13 |
| IC reason for testing at own HIV diagnosis (ref=no) | PWID | 1.36 | (0.88, 2.08) | 0.16 | 1.01 | (0.69, 1.48) | 0.96 |
|  | HIV+ partner | 2.08 | (1.32, 3.30) | <0.01 | 1.56 | (1.06, 2.31) | 0.02 |
|  | Pregnancy | 1.43 | (0.80, 2.57) | 0.23 | 1.09 | (0.76, 1.57) | 0.64 |
| IC HIV VL status (ref= VL<=1000) | Unsuppressed | 1.29 | (0.77, 2.16) | 0.33 | 2.68 | (1.81, 3.97) | <0.001 |
|  | VL not available | 1.06 | (0.61, 1.84) | 0.83 | 0.94 | (0.62, 1.41) | 0.76 |
| Partner age (continuous) | Each 5-year increase | 1.03 | (0.93, 1.14) | 0.58 | 1.09 | (1.00, 1.20) | 0.05 |
| Partner type (ref=sexual) | Needle-sharing | 1.08 | (0.56, 2.08) | 0.82 | 1.50 | (0.81, 2.77) | 0.19 |
|  | Child | 0.04 | (0.01, 0.12) | <0.001 | 0.37 | (0.14, 0.96) | 0.04 |
| Mode of partner notification (ref=client notification) | Provider or contract | 1.80 | (1.08, 3.02) | 0.03 | 1.48 | (0.96, 2.28) | 0.08 |
|  | Joint | 1.20 | (0.82, 1.77) | 0.35 | 0.79 | (0.55, 1.14) | 0.21 |

aOR=Adjusted odds ratio; CI=Confidence interval; IC=Index client; ART=Antiretroviral therapy; PWID=Person with current or past injection drug use; MSM=Man having sex with men; VL=HIV viral load; ^*^Excludes 27 partners with IPV concern reported.

^* *^ Provider and contract notification modes combined due to small cell size for contract mode (n=25). Results based on mixed effects logistic regression models, which treated health facility and ICs as random effects (to address nesting of observations on partners within health facilities and ICs).
